# Apelin-13 Inhibits Large-Conductance Ca^2+^-Activated K^+^ Channels in Cerebral Artery Smooth Muscle Cells via a PI3-Kinase Dependent Mechanism

**DOI:** 10.1371/journal.pone.0083051

**Published:** 2013-12-26

**Authors:** Amit Modgil, Lirong Guo, Stephen T. O’Rourke, Chengwen Sun

**Affiliations:** 1 Department of Pathophysiology, College of Basic Medical Sciences, Jilin University, Changchun, Jilin, China; 2 Department of Pharmaceutical Sciences, North Dakota State University, Fargo, North Dakota, United States of America; Xuzhou Medical college, China

## Abstract

Apelin-13 causes vasoconstriction by acting directly on APJ receptors in vascular smooth muscle (VSM) cells; however, the ionic mechanisms underlying this action at the cellular level remain unclear. Large-conductance Ca^2+^-activated K^+^ (BK_Ca_) channels in VSM cells are critical regulators of membrane potential and vascular tone. In the present study, we examined the effect of apelin-13 on BK_Ca_ channel activity in VSM cells, freshly isolated from rat middle cerebral arteries. In whole-cell patch clamp mode, apelin-13 (0.001-1 μM) caused concentration-dependent inhibition of BK_Ca_ in VSM cells. Apelin-13 (0.1 µM) significantly decreased BK_Ca_ current density from 71.25±8.14 pA/pF to 44.52±7.10 pA/pF (n=14 cells, P<0.05). This inhibitory effect of apelin-13 was confirmed by single channel recording in cell-attached patches, in which extracellular application of apelin-13 (0.1 µM) decreased the open-state probability (NP_o_) of BK_Ca_ channels in freshly isolated VSM cells. However, in inside-out patches, extracellular application of apelin-13 (0.1µM) did not alter the NP_o_ of BK_Ca_ channels, suggesting that the inhibitory effect of apelin-13 on BK_Ca_ is not mediated by a direct action on BK_Ca_. In whole cell patches, pretreatment of VSM cells with LY-294002, a PI3-kinase inhibitor, markedly attenuated the apelin-13-induced decrease in BK_Ca_ current density. In addition, treatment of arteries with apelin-13 (0.1 µM) significantly increased the ratio of phosphorylated-Akt/total Akt, indicating that apelin-13 significantly increases PI3-kinase activity. Taken together, the data suggest that apelin-13 inhibits BK_Ca_ channel via a PI3-kinase-dependent signaling pathway in cerebral artery VSM cells, which may contribute to its regulatory action in the control of vascular tone.

## Introduction

 Apelin is a peptide isolated from bovine stomach extracts and identified as an endogenous ligand for the orphan G-protein coupled receptor, APJ, which has seven transmembrane-spanning domains [1,2]. Despite sharing 31% of amino acid sequence homology with angiotensin II type 1 receptors (AT1-R), angiotensin II does not bind to these receptors [3] and apelin is the only known ligand for APJ receptors. The apelin gene, located on the long arm of the human X chromosome, codes for a preproprotein of 77-amino acid residues that is further cleaved to shorter active peptide fragments including apelin-36 (42-77), apelin-17 (61-77) and apelin-13 (65-77) [1,4,5]. Apelin-13 exhibits the greatest binding affinity and biological potency as compared to other fragments [4,6,7]. Apelin is expressed in the brain cardiovascular regulatory areas as well as in peripheral tissues including the heart and vasculature of major organs, such as lung, kidney and adrenal gland [4,8-11]. Moreover, APJ receptors are expressed in VSM cells and cardiomyocytes [12]. The wide expression pattern of apelin/APJ receptors throughout the cardiovascular system strongly suggests an important role for the apelin/APJ system in cardiovascular homeostasis. 

 In the cardiovascular system, apelin and APJ are expressed in endothelium, VSM and cardiomyocytes [12,13]. The vascular actions of apelin in regulating blood pressure and vascular tone are controversial. Bolus intravenous injection of apelin in rats causes a rapid and transient fall in mean atrial pressure [2,7,14]; in contrast, apelin induces contraction of isolated human saphenous veins and mammary arteries denuded of endothelium [12,13]. The latter studies are consistent with APJ expression on VSM cells [12]. Collectively, these data suggest that apelin peptides may have a biphasic hemodynamic response via endothelium-dependent vasorelaxation and a direct contractile effect on VSM. Apelin-induced endothelium-dependent vasorelaxation is attenuated by coadministration of L-NAME, a nitric oxide synthase inhibitor, suggesting an NO-dependent mechanism [7]. However, the intracellular transduction pathways and the molecular mechanisms underlying apelin-induced contractile response by direct action on VSM cells remain to be clarified.

 Cerebral arteries express several types of K^+^ channels [17]. These include large conductance calcium activated K^+^ (BK_Ca_) channels, which are highly expressed in VSM cells and play an essential role in regulating resting membrane potential and, hence, vascular tone [17,18]. Activation of BK_Ca_ channels in smooth muscle leads to efflux of K^+^ from the cell and causes hyperpolarization, which decreases the activity of voltage-gated L-type Ca^2+^ channels and subsequently leads to vasorelaxation. BK_Ca_ channel inhibition causes depolarization, increasing the activity of voltage gated L-type Ca^2+^ channels and subsequently leads to vasoconstriction [19]. BK_Ca_ channel activity is controlled by multiple factors, including intracellular calcium levels, phosphorylation status, and oxidation state [18]. At present, the interaction between apelin and BK_Ca_ channels in VSM cells is not fully understood.

 The purpose of the present study was to determine the effect of apelin-13 on BK_Ca_ channel activity and to investigate the intracellular signaling mechanisms by which apelin regulates BK_Ca_ channels in VSM cells freshly isolated from rat middle cerebral arteries. Our results indicate that apelin-13 inhibits BK_Ca_ channel activity in cerebral arterial VSM cells and that the inhibitory action of apelin-13 is mediated by a G-protein and PI3-kinase-dependent signaling pathway. 

## Methods

### Animals and chemicals

 Experiments were performed on 12-week-old male Sprague-Daley (SD) rats purchased from Charles River Farms (Wilmington, MA). Rats were housed at 22 ± 2°C on a 12 h-12 h light-dark cycle and provided with food and water ad libitum. All animal protocols were approved by the North Dakota State University Institutional Animal Care and Use Committee. 

 Crystallized papain, collagenase and elastase were purchased from Worthington Biochemicals (Freehold, NJ). Rabbit anti-Akt and anti-p-Akt antibodies were purchased from Santa Cruz Biotechnology (Santa Cruz, CA). Rabbit anti-APJ receptor antibodies were purchased from Abcam Inc. (Cambridge, MA). Anti-rabbit peroxidase-conjugated antibody was purchased from Bio-Rad (Hercules, CA). Anti-alpha-actin antibodies, pertussis toxin, iberiotoxin, LY-294002, soybean trypsin inhibitor, DTT, ATP, GTP, HEPES, and other reagents were obtained from Sigma-Aldrich (St. Louis, MO).

### Western blot analysis

 Akt and p-Akt protein levels in rat cerebral arteries were assessed by Western blot analysis as described previously [20]. Male SD rats (BW: 200 to 300 g; n=6) were euthanized with an excessive dose of pentobarbital sodium. The cerebral arteries from the whole brain were dissected and immediately put in ice cold Tyrode's solution containing (in mM) 145 NaCl, 4 KCl, 0.05 CaCl_2_, 1 MgCl_2_, 10 HEPES, 10 dextrose; pH 7.4 (NaOH). After washing with ice cold Tyrode's solution, the cerebral arteries were treated with or without apelin-13 (0.1 µM) for 5, 10, 15, or 30 min. To test the effect of LY-294002 on the effect of apelin on PI3-kinase activity, arteries were treated with apelin-13 plus LY-294002 (10 µM) for 15 minutes. Vascular tissues were then merged in liquid nitrogen, mashed, and homogenized in the lysis buffer. An aliquot of 20 µg of protein from each sample was separated on a 10% SDS-PAGE gel and transferred onto nitrocellulose membranes for 2 h at 100 V. After a 10-min wash in PBS-T, membranes were blocked in PBS-T containing 10% milk for 1 h, followed by overnight incubation with rabbit anti-Akt or rabbit Anti p-Akt antibody (dilution 1:500) at 4°C. After a 15-min wash in PBS-T, four 5-min washes in PBS-T were carried out, and membranes were incubated for 2h in an anti-rabbit peroxidase-conjugated antibody (dilution 1:15,000). Densitometry of p-Akt was normalized to Akt. Immunoreactivity was detected by enhanced chemiluminescence autoradiography (ECL Western blotting detection kit, Amersham Pharmacia Biotechnology), and film was analyzed with Quantity One Software (Bio-Rad).

### Isolation of VSM cells from rat middle cerebral arteries

 Enzymatic isolation of single VSM cells was carried out as previously described [21]. Briefly, brain tissues were rapidly removed and placed in 4°C cold Tyrode's solution containing (in mM) 145 NaCl, 4 KCl, 0.05 CaCl_2_, 1 MgCl_2_, 10 HEPES, 10 dextrose; pH 7.4 (NaOH). Middle cerebral arteries with small branches were dissected and cleaned. The vessel segments were incubated for 15 minutes at 37°C in 1 ml of low Ca^2+^ Tyrode's solution containing 1.5 mg/ml papain (14 U/mg) and 1 mg/mL DTT, followed by incubation for 15 minutes at 37°C in 2 mL of Tyrode's solution containing 2 mg/mL collagenase (196 U/ml), 0.5 mg/mL elastase (90 U/ml), and 1 mg/mL soybean trypsin inhibitor (10 000 U/ml). The supernatant was collected and the cells spun down at 500 *g* for 5 minutes, re-suspended in fresh low Ca^2+^ Tyrode's solution, and stored at 4°C. Patch-clamp experiments were completed within 4 hours after the cells were isolated. To identify VSM cells and confirm APJ receptor expression in VSM cells, the isolated cells were immunostained with VSM specific alpha-actin antibodies and APJ receptor antibodies as detailed in our previous publication [11].

### Electrophysiological recordings

 Patch clamp recording techniques were used to measure currents in the whole cell, cell-attached or inside-out patch clamp configurations. The patch electrodes were fabricated from 1.5-mm borosilicate glass capillaries and filled with prefiltered solutions of different composition (see below). The currents were recorded at room temperature. Voltage-clamp and voltage-pulse generation were controlled with an Axopatch 200B patch-clamp amplifier (Axon Instruments, Burlingame, CA). Current data were collected and analyzed with pCLAMP 10.0 software (Molecular Devices). Voltage-activated currents were filtered at 2 kHz and digitized at 10 kHz, and capacitative and leakage currents were subtracted digitally. All drugs were diluted in fresh bath solution and perfused into a 35mm nunc cell culture dishes. Series resistance and total cell capacitance were calculated from uncompensated capacitive transients in response to 10 ms hyperpolarizing step pulses (5 mV), or obtained by adjusting series resistance and whole-cell capacitance using the Axopatch 200B amplifier control system.

 Whole cell BK_Ca_ currents in VSM cells were recorded using the whole-cell configuration of the patch clamp technique. VSM cells were superfused at a rate of 2.0 ml/min with a solution containing (in mM) 145 NaCl, 5.4 KCl, 1.8 CaCl_2_, 1 MgCl_2_, 5 HEPES, 10 dextrose; pH 7.4 (NaOH). The recording pipettes had resistances of 3 to 4 M; and were filled with a solution containing (in mM) 145 KCl, 5 NaCl, 0.37 CaCl_2_, 2 MgCl_2_, 10 HEPES, 1 EGTA, 7.5 Dextrose; pH 7.2 (KOH). Standard recording conditions for BK_Ca_ were achieved by stepping from a holding potential of –70 to +50 mV by stepping 10 mV increments. BK_Ca_ was expressed as current density (current divided by its capacitance), and all recordings were performed at room temperature. Other potassium channel currents were ruled out by subtraction from total currents with the currents recorded in the presence of iberiotoxin, a specific BK_Ca_ blocker, at the end of each protocol. 

 Single BK_Ca_ channel currents were measured in cell-attached patches and inside-out excised patches in VSM cells as described in our previous publication [21]. The recording pipettes (4–5 MΩ) were filled with a solution containing (in mM): 145 KCl, 1.8 CaCl_2_, MgCl_2_ 1.1, and 5 HEPES; pH 7.2 (KOH). Freshly isolated VSM cells were bathed in a recording chamber filled with a solution containing (in mM): 145 KCl, 1.1 MgCl_2_, 0.37 CaCl_2_, 10 HEPES, 1 EGTA and 10 glucose; pH 7.4 (KOH). The results were expressed as open-state probability (NP_o_). The NP_o_ calculation and BK_Ca_ channel characterization were performed as described previously [21]. 

### Data analysis

 Results are expressed as means ± SE. Statistical significance was evaluated by one-way or two-way ANOVA, as appropriate, followed by either a Newman-Keuls or Bonferroni post hoc analysis where appropriate. Differences were considered significant at P < 0.05, and individual probability values are noted in the figure legends. 

## Results

### Effect of Apelin-13 on BK_Ca_ channel currents in VSM cells

**Figure 1:**
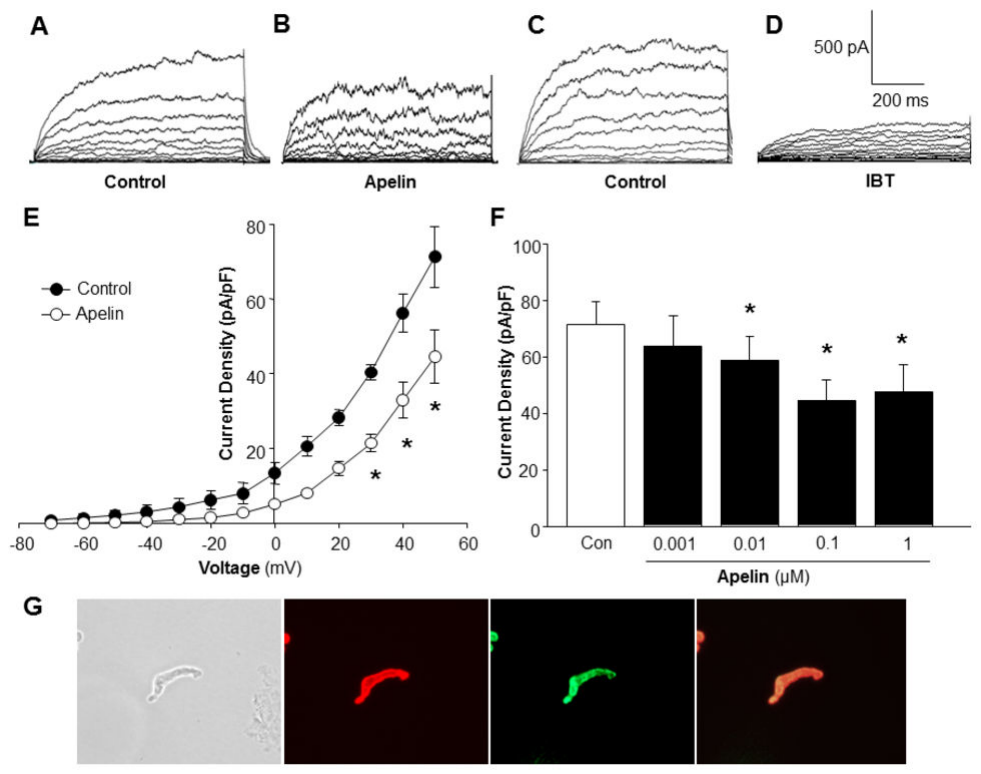
Effect of apelin-13 on activity of large conductance Ca^2+^-activated K^+^ (BK_Ca_ ) channels of rat middle cerebral arterial VSM cells. Whole-cell K^+^ currents were recorded at room temperature in response to successive voltage pulses of 800 ms duration, increasing in 10-mV increments from -70 mV to +50 mV before and after the treatment of apelin-13 (0.001-1 µM). **A**-**D**: representative tracings depicting the currents recorded from a single VSM cell before and after treatment with apelin-13 (0.1 µM, 5 min) or iberiotoxin (IBT) (100 nM, 5 min). **E**: I-V curve plots of BK_Ca_ currents at baseline and after application of apelin-13 (0.1 µM, 5 min). **F**: Bar graph summarizing the concentration-dependent effect of apelin-13 (0.001-1 µM) on average current density (pA/pF) at +50 mV. Values are mean±SEM (n=6 to 14 cells). *P<0.05 indicates a significant difference from the corresponding control value. **G**: A representative VSM cell isolated from rat middle cerebral arteries under optical phase and fluorescence imaging. Fluorescence micrographs demonstrate the APJ receptor expression in VSM cells immunostained with anti-alpha smooth muscle actin antibodies (red) and anti-APJ receptor antibodies (green). The overlap of these two images shows that green fluorescence is VSM cell-located.

The effect of apelin-13 on BK_Ca_ channel activity was determined in VSM cells freshly isolated from rat middle cerebral artery. Whole-cell BK_Ca_ currents were recorded at room temperature and in response to successive voltage pulses of 800 ms duration, increasing in 10-mV increments from -70 mV to +50 mV in the absence or presence of apelin-13 (0.1 µM). Superfusion with apelin-13 (0.1 µM, 5 min) significantly reduced BK_Ca_ current density from 71.25±8.14 pA/pF to 44.52±7.10 pA/pF (n=14 cells, P<0.05), as depicted in Figure 1. In addition, the selective BK_Ca_ channel blocker, iberiotoxin, markedly attenuated most of the whole-cell current in cerebral artery VSM cells under these conditions (Figure 1C and D). The results demonstrate that apelin-13 inhibits BK_Ca_ current density in cerebral artery VSM cells in a concentration-dependent manner, beginning at 0.01 µM and reaching a maximum effect at 0.1µM (Figure 1F). In this study, we also determined the time-course of apelin-13 action on BK_Ca_ current density in VSM cells. The response to apelin-13 was rapid, reaching a peak in 5 min, and lasting at least 30 min. The expression of APJ receptors in VSM cells was confirmed by immunocytochemistry study using VSM cell specific anti-alpha-actin antibodies and anti-APJ receptor antibodies (Figure 1G). 

### Effect of pertussis toxin on the inhibitory action of apelin-13 on BK_Ca_ channel activity

**Figure 2:**
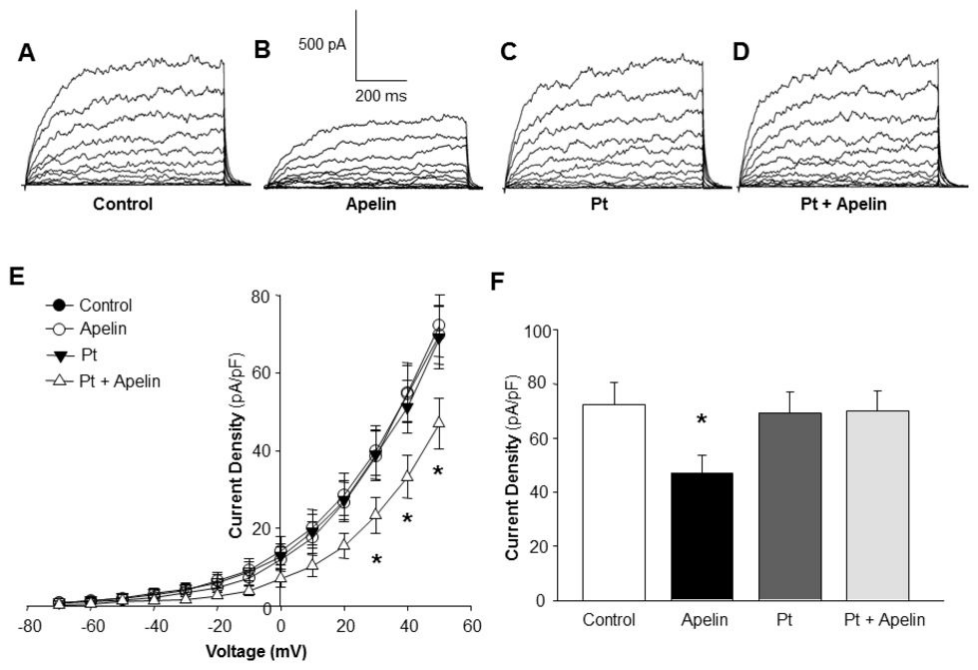
Effect of pertussis toxin and apelin-13 on BK_Ca_ channels in rat middle cerebral arterial VSM cells. Whole-cell BK_Ca_ currents were recorded in rat middle cerebral arterial VSM cells as described in the Methods. **A**-**D**: representative tracings depicting the currents recorded from a single VSM cell under the following sequential treatment conditions: Control (A), followed by superfusion with apelin-13 (0.1 µM, 5 min) (B), washout of apelin-13, superfusion with Pertussis toxin (PT, 100 nM, 5 min) (C), and superfusion with apelin-13 plus Pertussis toxin (D). **E**: Average whole-cell current-voltage plots of BK_Ca_ current. **F**: Bar graphs summarizing the average current density (pA/pF) at +50 mV obtained at each treatment condition described above. Values are mean±SEM (n=6). *P<0.05 indicates a significant difference from the corresponding control value.

 It was previously reported that APJ receptors expressed in Chinese hamster ovary cells are coupled to pertussis toxin-sensitive G proteins (Gi/Go protein) (22, 23). To further investigate the signaling mechanisms that lead to inhibition of BK_Ca_ channel activity, the effect of pertussis toxin on apelin-13-induced inhibition of BK_Ca_ in cerebral VSM cells was determined. The results presented in Figure 2 demonstrate that pre-treatment of VSM cells with pertussis toxin (100 nM) completely abolished the inhibitory effect of apelin-13, suggesting that the inhibitory effect of apelin-13 on BK_Ca_ channels is mediated by APJ receptors that are functionally coupled to Gi/Go proteins.

### Effects of apelin-13 on BK_Ca_ channel activity in cell-attached patches of VSM cells

**Figure 3:**
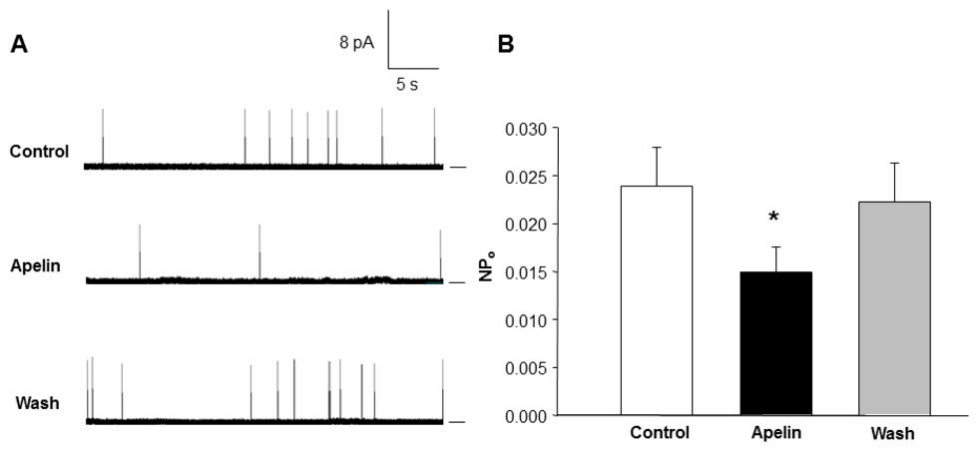
Effects of apelin-13 on the activity of BK_Ca_ channels recorded from cell-attached patches of rat middle cerebral arterial VSM cells. Currents were recorded at room temperature with a pipette potential of -40 mV. **A**: Representative tracings showing the large-conductance K^+^ channel currents recorded from cell-attached patches of VSM cells under the following sequential treatment conditions: Control; apelin-13 (0.1 µM, 5 min); washout. **B**: Bar graph summarizing the open state probability (NP_o_) of BK_Ca_ channels during each treatment condition described above. *P<0.05 indicates a significant difference from the corresponding control value. Values presented are mean±SEM recorded from 9 cells.

 The inhibitory effect of apelin-13 on BK_Ca_ channels was confirmed using single channel recording in cell-attached patches from VSM cells. Single channel recording was performed on cells bathed in a high K^+^ solution to control the membrane potential. Treatment of VSM cells with apelin-13 significantly inhibited the activity of a large conductance channel that carries an outward current. As shown in Figure 3, apelin-13 (0.1 µM) significantly reduced BK_Ca_ channel activity by 38%. The probability of channel opening (NP_o_) was decreased from 0.0239 ±.00408 to 0.0148 ± 0.00267 (n=9, P<0.05); however, apelin -13 did not alter unitary conducance of this channel. The inhibitory effect of apelin-13 on BK_Ca_ channel activity was readily reversible upon washout (Figure 3). 

### Effects of apelin-13 on BK_Ca_ channel activity in inside-out patches of VSM cells

**Figure 4:**
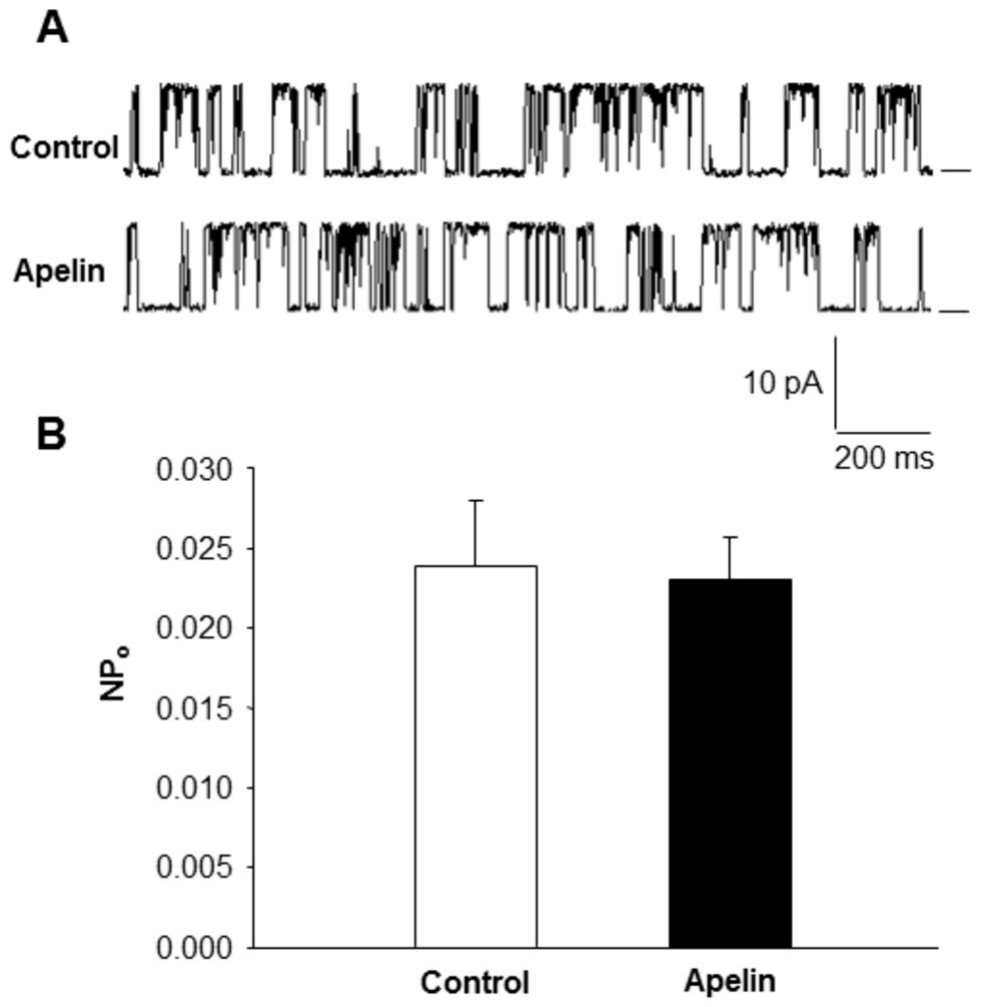
Effect of apelin-13 on BK_Ca_ channel activity recorded from inside-out patches of VSM cells freshly isolated from rat middle cerebral arteries. Currents were recorded at room temperature before and after administration of apelin-13 through the recording pipettes. **A**: Representative tracings depicting the effects of apelin-13 (0.1 µM, 5 min) on the large-conductance K^+^ channel currents recorded from inside-out patches of VSM cells isolated from rat middle cerebral arteries. **B**: Bar graph summarizing the open state probability (NP_o_) of BK_Ca_ channels before and after administration of apelin-13 (0.1 µM). Values presented are mean±SEM recorded from 7 cells.

 To investigate whether apelin directly inhibits the BK_Ca_ channel, BK_Ca_ channel activity was recorded in inside-out patches of VSM cells before and after application to the recording pipettes. BK_Ca_ channel activity was measured in excised inside-out membrane patches of VSM cells. The results are presented in Figure 4, demonstrating that extracellular application of apelin-13 did not alter BK_Ca_ open state probability. This observation suggests that the inhibitory action of apelin-13 is mediated by an intracellular signaling pathway rather than acting directly on the channel. Therefore, we performed the following experiments to identify the intracellular signaling pathway underlying the apelin-13-induced inhibitory action on BK_Ca_ channels in VSM cells. 

### Effect of blockade of PI3-kinase on the action of apelin-13 on BK_Ca_ channel activity

**Figure 5:**
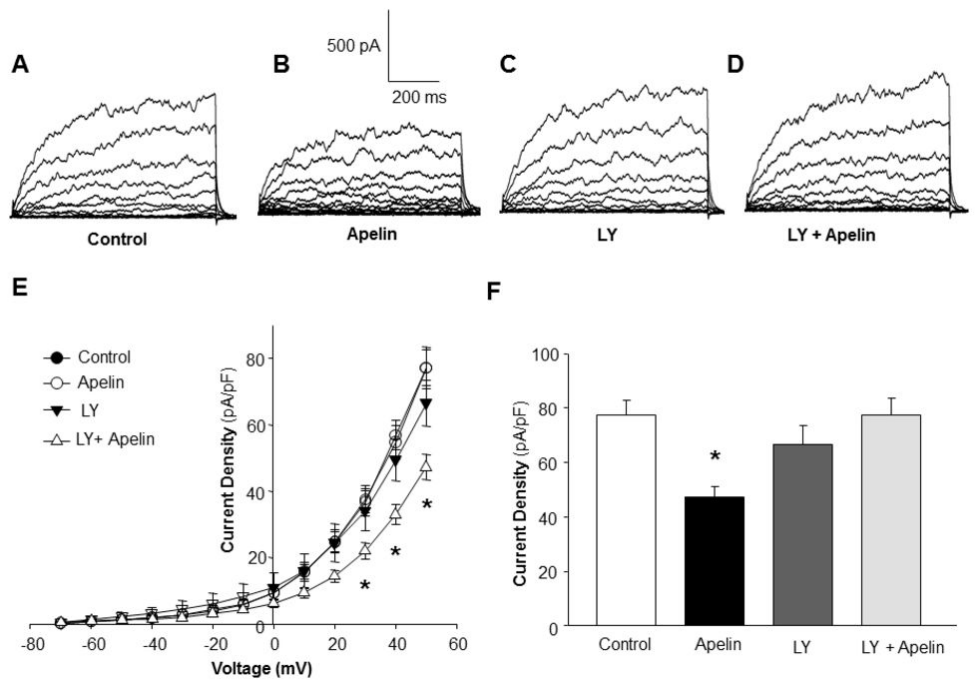
Effect of PI3-kinase inhibition on the inhibitory action of apelin-13 on BK_Ca_ channel currents in rat middle cerebral arterial VSM cells. Whole-cell BK_Ca_ currents were recorded as described in the Methods. **A**-**D**: Representative tracings showing the currents recorded from a single VSM cell under the following sequential treatment conditions: control (A); followed by superfusion with 0.1 µM apelin-13 (B) for 5 min; washout of apelin-13; superfusion with LY-294002 (10 mM, 5 min) (C); and superfusion with apelin-13 plus LY-294002 (D). **E**: Average whole cell current density-voltage plots of BK_ca_ current. **F**: Bar graph summarizing the average current density (pA/pF) at 50 mV obtained at each treatment condition described above. Values are mean±SEM (n=6 cells). *P<0.05 indicates a significant difference from the corresponding control value.

 It has been previously demonstrated that apelin-13 stimulates phosphatidylinositol 3-kinase (PI3-kinase) in VSM cells [23]. Thus, we examined the effect of apelin-13 on BK_Ca_ channels with and without pretreatment with LY-294002, a selective PI3-kinase inhibitor, in cerebral VSM cells. The whole-cell BK_ca_ current was recorded under control conditions, and in the presence of apelin-13 (0.1 µM, 5 min) alone, LY-294002 (10 µM, 5 min) alone, or LY-294002 plus apelin-13. Treatment of VSM cell with LY-294002 alone did not alter BK_Ca_ channel activity; however, pretreatment of VSM cells with LY-294002 significantly attenuated the inhibitory effect of apelin-13 by 96% (Figure 5). These results demonstrate that PI3-kinase may play a role in the apelin-13-induced reduction of BK_ca_ channel activity in VSM cells.

### Effects of Apelin-13 and LY-294002 on PI3-kinase activity in cerebral arteries

**Figure 6:**
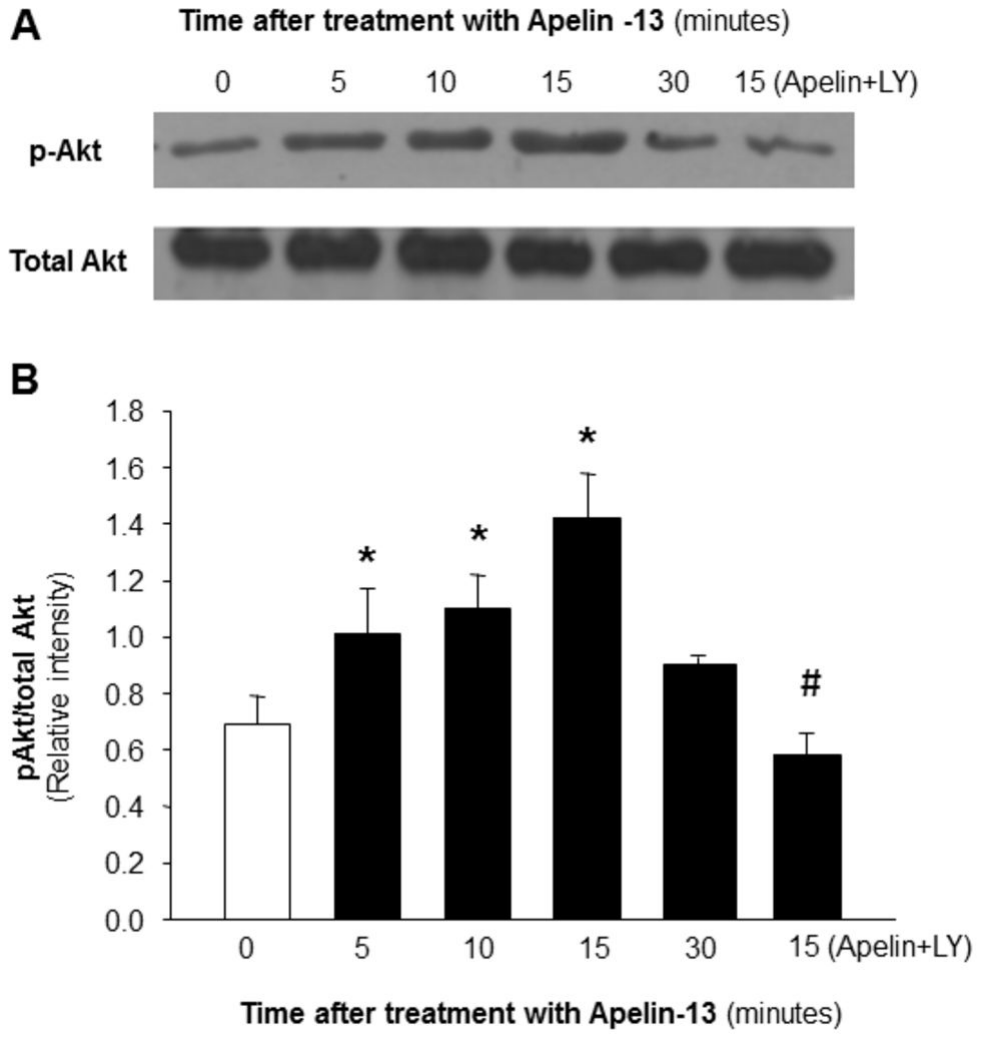
Effect of apelin-13 on PI3-kinase activity measured in rat cerebral arteries using western blot as described in the Methods. Phosphorylated AKT and total AKT were measured in cerebral arteries treated with apelin-13 (0.1 µM) or apelin-13 plus LY-294002 (LY, 10 µM) for durations indicated in the figure. **A**: The represented blots were probed with anti serine-phosphorylation of Akt (pAkt) or anti-Akt (total) antibodies. **B**: Bar graphs summarizing the effect of apelin-13 on PI3-kinase activity expressed as a ratio of p-Akt/Akt protein levels. Data are means ± SEM derived from three independent experiments. *P<0.05 compared with non-stimulated control.

PI3-kinase activity was detected by the ratio of phosphorylated Akt and total Akt using Western blots in cerebral arteries treated under control conditions and in the presence of apelin-13 (0.1 µM) alone or apelin-13 plus LY-294002 (10 µM). Apelin-13 elicited a time-dependent increase in phosphorylation of Akt, which peaked at 15 minutes (Figure 6). Treatment with apelin-13 (0.1 µM, 15 min) induced a two-fold increase in PI3-kinase activity. The inhibitory effect of apelin-13 was completely blocked by coincubation with LY-294002. These results demonstrate that apelin-13 stimulates PI3-kinase activity in cerebral arteries, suggesting that PI3-kinase may be involved in the action of apelin-13 in VSM cells. 

## Discussion

 The present study was undertaken to examine the effect of apelin-13 on BK_ca_ channel activity in VSM cells freshly isolated from rat middle cerebral arteries. The results demonstrate that apelin-13 inhibits BK_Ca_ channel activity in a concentration-dependent manner through G-protein and PI3-kinase dependent signaling pathways, which may contribute to its regulatory action in controlling vascular tone. This conclusion is supported by the following pieces of evidence: (1) apelin-13 inhibits BK_Ca_ current density in whole cell patches and BK_Ca_ channel activity in cell-attached patches in VSM cells freshly isolated from rat middle cerebral arteries; (2) apelin-13 did not significantly alter the open state probability of BK_ca_ channels in excised inside-out membrane patches; (3) the inhibitory effect of apelin-13 was abolished in the presence of a G-protein inhibitor or PI3-kinase inhibitor; and (4) apelin-13 significantly increased PI3-kinase activity in cerebral arteries.

Apelin induces vasodilation in isolated human mesenteric and mammary arteries with intact endothelium, an effect that is abolished by nitric oxide synthase inhibitors [7,25]. These studies suggest that apelin may act on endothelial APJ receptors to generate nitric oxide, which diffuses to underlying VSM cells and produces vasodilation. In contrast, apelin causes contraction in endothelium-denuded human saphenous veins and mammary arteries [15,16]. These studies indicate that apelin elicits both vasoconstrictor and vasodilator responses, either by a direct action on VSM cells or by an indirect effect mediated via endothelial cells, respectively. Although the mechanism of the endothelium-dependent vasodilator effect of apelin is well studied, the cellular mechanism of the direct action of apelin on VSM cells is not fully understood. The present study provides important new evidence that apelin acts directly on VSM cells to inhibit BK_Ca_ channels, which could contribute to the vasoconstrictor action of apelin in arteries. 

 Although multiple classes of potassium channels are expressed at varying densities in different vascular beds, the large conductance, calcium- and voltage- activated potassium (BK_Ca_) channel is the predominant K^+^ channel present in most arteries and plays an essential role in the regulation of vascular tone [17,18]. In the current study, apelin-13 inhibited the whole cell BK_ca_ channel current density in a concentration-dependent manner. This action of apelin-13 was confirmed in cell-attached patches in cerebral VSM cells isolated from rat middle cerebral artery. Considering previous observations showing that apelin-13 induces vasoconstriction in endothelium-denuded arteries and veins, we anticipate that the direct inhibitory effect of apelin-13 on BK_Ca_ channel activity in VSM cells may contribute to the vasoconstrictor effect of this regulatory peptide. However, this interpretation still needs further investigation. 

 The inhibitory effect of apelin on BK_Ca_ channels was absent in patches excised from the cellular membrane, suggesting that this action of apelin is mediated by intracellular signaling molecules. Thus, we performed several experiments to identify the signaling pathways mediating this effect of apelin. Previous studies demonstrated that APJ receptor immunoreactivity was observed in human and rat VSM cells [12,13], and that APJ is a G protein coupled receptor. G proteins also are involved in several actions of apelin, such as a positive inotropic effect in the heart [14,22,23,26]. Consistent with these studies, we demonstrated that the inhibitory effect of apelin-13 on BK_Ca_ channels was blocked by pertussis toxin, indicating that G_i_ protein is also involved in the action of apelin in VSM cells. We also observed that basal levels of Akt phosphorylation were increased after preincubation of arteries with apelin-13 and that this effect was inhibited in the presence of LY-294002, a PI3-kinase inhibitor, suggesting the involvement of PI3-kinase/Akt as a downstream signaling pathway in the signal transduction cascades of apelin-13. This stimulatory effect of apelin on PI3 kinase was reported by another research group in VSM cells [24] and was also observed in other tissues [14,23]. Since PI3-kinase may be involved in the regulation of BK_Ca_ channel activity [27], we studied the role of PI3-kinase in the action of apelin on BK_Ca_ channels in VSM cell. In whole cell recording studies, the inhibitory effect of apelin-13 on the BK_Ca_ current was significantly blocked by LY-294002, a selective inhibitor for PI3-kinase, thus providing further evidence for a role for PI3-kinase in the response to apelin. However, the downstream signaling pathway involved in PI3-kinase mediated inhibition of BK_Ca_ channels is not yet clear. One possibility is that this action of PI3-kinase is mediated by a membrane lipid, phosphatidylinositol 4,5-bisphosphate (PIP2), that is produced by PI3-kinase. This hypothesis is supported by an observation in inside-out patches of VSM cells from cerebral arteries, showing that direct addition of PIP2 onto the inner surface of the cytoplasmic membrane significantly increase open probability of BK_Ca_ channels in VSM cells [28]. On the other hand, a recent study from Gebremedhin and his colleagues demonstrates that the regulation of BK_Ca_ channels by PI3-K could be mediated by Akt-dependent phosphorylation of BK_Ca_ channels in cerebral VSM cells [29]. Thus, the exact signaling mechanisms underlying PI3-kinase-induced inhibition of BK_Ca_ channels in the action of apelin-13 still need further investigation. 

 In conclusion, we have demonstrated that apelin-13 directly acts on VSM cells and inhibits BK_Ca_ channel activity. The inhibitory effect of apelin on BK_Ca_ channels is mediated by a G-protein and PI3-kinase dependent signaling pathway. This action of apelin in VSM cells could contribute to its regulatory action in control of vascular tone.
